# Integrative Analysis of TP53INP2 in Head and Neck Squamous Cell Carcinoma

**DOI:** 10.3389/fgene.2021.630794

**Published:** 2021-04-09

**Authors:** Ruoyan Cao, Suyang Liu, Jiayu Zhang, Xianyue Ren, Xijuan Chen, Bin Cheng, Juan Xia

**Affiliations:** ^1^Hospital of Stomatology, Sun Yat-sen University, Guangzhou, China; ^2^Guangdong Provincial Key Laboratory of Stomatology, Guangzhou, China; ^3^Guanghua School of Stomatology, Sun Yat-sen University, Guangzhou, China

**Keywords:** TP53INP2, head and neck squamous cell carcinoma, prognosis, bioinformatics, multi-omics

## Abstract

TP53INP2 plays an important role in regulating gene transcription and starvation-induced autophagy, however, its function in head and neck squamous cell carcinoma (HNSCC) remains unclear. Therefore, we assessed the expression and prognostic value of TP53INP2. In addition, RNAseq, miRNAseq, copy number variation, and mutation profiles from The Cancer Genome Atlas (TCGA) dataset were applied to evaluate the distinctive genomic patterns related to TP53INP2 expression. We found that TP53INP2 expression was lower in HNSCC compared with normal controls. Patients with higher TP53INP2 expression had longer survival time. Knockdown of TP53INP2 promoted cell viability. Functional analysis exhibited that TP53INP2 was linked to DNA replication, DNA repair, cell cycle, and multiple metabolic pathways. Moreover, TP53INP2 might affect the expression of multiple genes *via* enhancing the transcriptional activity of nuclear hormone receptors. A competing endogenous RNA (ceRNA) network consisting of 33 lncRNAs, eight miRNAs, and 13 mRNAs was constructed based on the expression of TP53INP2. Taken together, our study highlights the potential value of TP53INP2 in predicting the survival of HNSCC and its important role in the genesis and development of HNSCC.

## Introduction

Head and neck squamous cell carcinoma (HNSCC) is the sixth most prevalent cancer with approximately 830,000 new cases and 430,000 related death per year worldwide, bringing a heavy burden to the patients and the society (Bray et al., 2018; Yu et al., 2019). Despite of the great advancements in surgical excision, chemotherapy, and radiotherapy in the past years, the 5-year overall survival rate of HNSCC has not been effectively improved, about 50% (van der Schroeff et al., 2010). Thus, there is an urgent need to further understand the oncogenesis and development of HNSCC as well as developing novel therapeutic targets.

Tumor protein p53-inducible nuclear protein 2 (TP53INP2), also named diabetes- and obesity-related gene (DOR), is a bi-functional protein that is associated with gene transcription and starvation-induced autophagy (Xu and Wan, 2020). In detail, TP53INP2 is a nuclear protein serving as a transcriptional coactivator of nuclear hormone receptors, including thyroid hormone receptor alpha (THRA/TRα), glucocorticoid receptor (GR), vitamin D receptor (VDR), and peroxisome proliferator-activated receptor gamma (PPARG) (Baumgartner et al., 2007; Sancho et al., 2012). Under starvation and rapamycin treatment, nuclear TP53INP2 moves to the cytoplasm and involves in autophagy (Nowak et al., 2009; Huang et al., 2015; You et al., 2019). In addition, TP53INP2 regulates rDNA transcription and interacts with ubiquitinated proteins and ubiquitin (Xu et al., 2016; Xu and Wan, 2019). In bladder cancer, TP53INP2 is upregulated and knockdown of TP53INP2 inhibits the invasion, migration as well as epithelial-to-mesenchymal transition (Zhou et al., 2020). However, the function of TP53INP2 in HNSCC is largely unclear.

In this study, we found TP53INP2 was down-regulated in HNSCC based on multiple datasets and validated these results in multiple HNSCC cell lines. Knockdown of TP53INP2 promoted CAL27 cell proliferation. We also investigated the functional network, genomic alterations, and competing endogenous RNA (ceRNA) network related to TP53INP2 in HNSCC. Our results highlight the potential value of TP53INP2 in the treatment of HNSCC.

## Materials and Methods

### Data Source

Level 3 RNA-Seq data and the corresponding clinical data for our HNSCC datasets were downloaded from the TCGA data portal^1^. After excluding the HNSCC patients followed for no more than 30 days and more than 2,000 days, we finally included 455 HNSCC patients and 44 normal controls for our subsequent analysis. Besides, we also downloaded five HNSCC datasets (GSE30784, GSE31056, GSE33205, GSE59102, and GSE33791) from Gene Expression Omnibus (GEO) database^2^ to validate the expression of TP53INP2 between HNSCC and normal controls.

### Gene Set Enrichment Analysis

We divided TCGA HNSCC cohort into two groups (TP53INP2-high and TP53INP2-low) based on the R package “survminer.” To explore the difference in biological process between the different expressions of TP53INP2, we performed Gene Set Enrichment Analysis (GSEA) using “clusterProfiler” R packages. The gene sets of “h.all.v6.2.-symbols” and “c2.cp.kegg.v7.1- symbols” were obtained from the MSigDB database for running GSEA analysis. An adjusted *P* value less than 0.05 was considered to be statistically significant.

### Relationship Between TP53INP2 and Nuclear Hormone Receptors Targeted Genes

TP53INP2 could enhance the transcriptional activity of nuclear hormone receptors. Thus, we extracted targeted genes of nuclear hormone receptors from the TRRUST database^3^ and assessed the relationship between these genes and TP53INP2 based on the spearman correlation test. FDR < 0.05 was considered to be statistically significant. Cytoscape was used to visualize the regulatory network.

### CeRNA Network Based on TP53INP2 Expression

We identified significant differential mRNAs (DEmRNAs), miRNAs (DEmiRNAs), and lncRNAs (DElncRNAs) between TP53INP2-high and TP53INP2-low using “DEseq2” R package according to the criteria of absolute fold change (FC) more than 1.5 and FDR less than 0.05. LncRNA-miRNA interactions were predicted based on the starBase database^4^. The interactions of miRNA-mRNA were predicted by miRTarBase, miRDB, and TargetScan. To enhance the reliability of this ceRNA network, only miRNA-targeted mRNAs exist in all three databases and DEmRNAs were included. We employed Cytoscape software to visualize the ceRNA network.

### Copy Number Variation and Mutation Analysis

The copy number variation (CNV) profiles of HNSCC were extracted from GDAC Firehose^5^ and separated into different groups based on the expression of TP53INP2. We applied GISTIC 2.0 to detect significant deletion or amplification alterations in the whole genome. GISTIC 2.0 is a biological program to identify somatic CNVs by assessing the amplitude and frequency of corresponding events. The somatic mutation data of HNSCC patients was downloaded *via* the TCGAbiolinks and the data was visualized by “maftools” R package.

### Cell Transfection

The small interfere RNA (siRNA) specifically targeting TP53INP2 (si-TP53INP2) and the negative control (si-NC) were purchased from GenePharma (Suzhou, China). The sequence of siTP53INP2 is 5′–3′ GCAUGUCCGUUUACGUCACdTdT and CCUGAAAUCUGAAGGGCUUdTdT. Lipofectamine 3000 (Invitrogen, Carlsbad, CA, United States) was used to transfect si-TP53INP2 and si-NC into HNSCC cell according to the manufacturer’s protocol suggested.

### Quantitative Real-Time PCR

Total RNA was extracted from HOK, CAL27, CAL33, HSC3, HSC6, SCC25, and UM1 cells using the RNA-quick purification kit (ESscience Biotech). RNA (1 μg) was reversed into cDNA using HiScript^®^ III RT SuperMix (Vazyme). The obtained cDNA product was used for PCR amplification. Primer sequences are as follows: TP53INP2, forward 5′-CCTGTTCCCTTATTCTTCATTCC-3′, and reverse 5′-ATTCCCTCCATCTTCTCCCT-3′; GAPDH, forward 5′-CTCCTCCTGTTCGACAGTCAGC-3′, and reverse 5′-CCCAATACGACCAAATCCGTT-3′.

### Cell Viability

The CAL27 cell line was seeded on 96-well plates at a final density of 1.5 × 10^3^ cells per well. Cell proliferation was assessed using Cell Counting Kit-8 (CCK-8, Beyotime, China).

### Statistical Analysis

For survival analysis, we employed the log-rank test to compare the difference using the Kaplan–Meier survival curve. Spearman correlation analysis was used to assess the linear relationship between different genes. Two-tailed Student’s *t*-tests, Wilcoxon rank sum test, and one-way ANOVA test were used for assessing differences between groups. Unless otherwise specified, a *p* value less than 0.05 was considered to be statistically significant. R software (version: 3.6.2) was employed to perform all statistical analyses.

## Results

### Low Expression of TP53INP2 Predicts Prognosis in HNSCC

**FIGURE 1:**
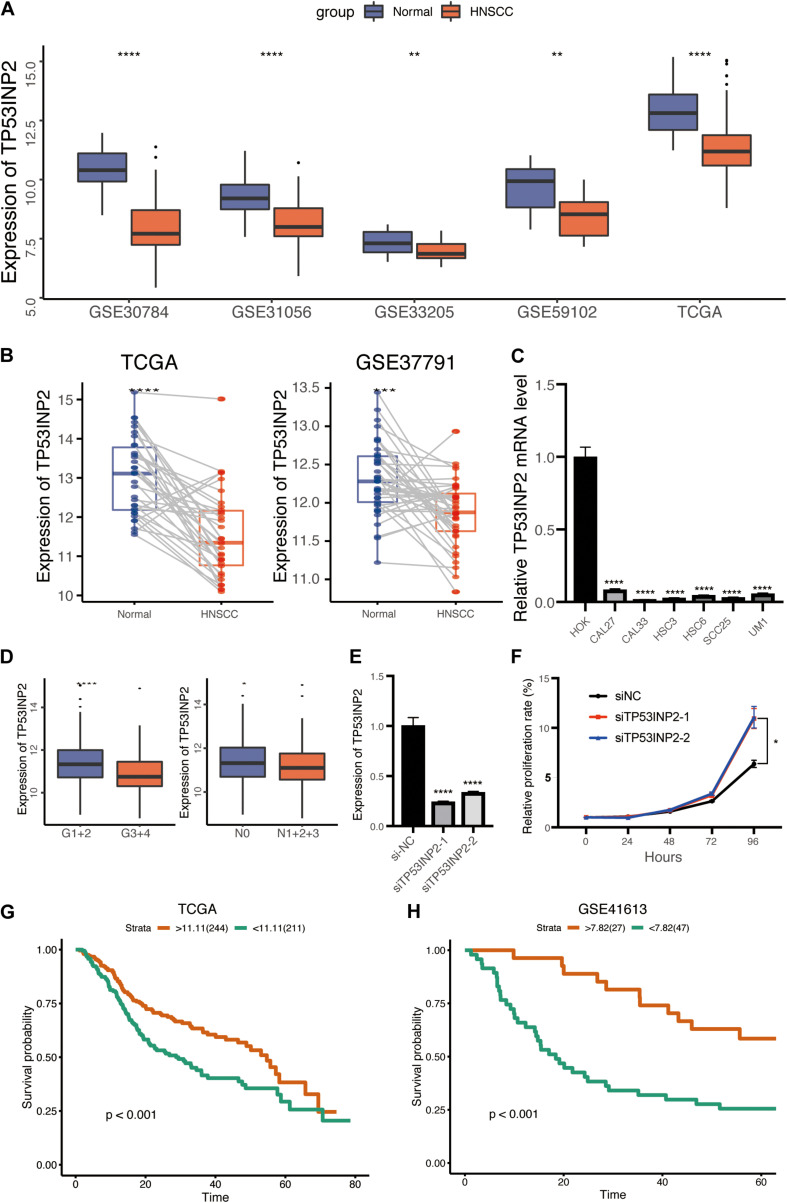
Low expression of TP53INP2 predicts prognosis in HNSCC. **(A)** Box plot of TP53INP2 expression between HNSCC and normal controls in multiple datasets. **(B)** Box plot of TP53INP2 expression between HNSCC and paired normal controls. **(C)** The relative expression of TP53INP2 expression between HNSCC cell lines and normal controls. **(D)** Box plot of TP53INP2 in grade and lymphatic metastasis subgroup. **(E)** TP53INP2 mRNA levels in CAL27 cell transfected with TP53INP2 siRNA. **(F)** Relative cell viability of CAL27 cell was measured by the CCK-8 at indicated time points. **(G,H)** Survival analysis of TP53INP2 in TCGA and GSE41613.

We assessed the expression of TP53INP2 in multiple HNSCC datasets from the GEO and TCGA. The results indicated that TP53INP2 was significantly downregulated in HNSCC than normal control (Figure 1A). Meanwhile, similar results were found in pairs of HNSCC and normal tissues (Figure 1B). Furthermore, the down-regulated expression of TP53INP2 in multiple HNSCC cell lines was validated using quantitative real-time PCR (qRT-PCR) (Figure 1C). We also found that TP53INP2 was significantly decreased in patients with poor differentiation and lymphatic metastasis (Figure 1D). To further verify the function of TP53INP2, the knockdown of TP53INP2 in CAL27 cell line was performed (Figure 1E). The subsequent CCK8 assay indicated that TP53INP2 knockdown significantly promoted cell proliferation (Figure 1F). According to the “survminer” R package, we determined the best cut-off point to stratify TCGA-HNSCC patients into TP53INP2-high (*n* = 224) and TP53INP2-low (*n* = 211) subgroups. Low expression of TP53INP2 was associated with poor prognosis (Figure 1G). In line with the results in TCGA cohort, we found that patients with lower expression in GSE41613 cohort also exhibited shorter survival time (Figure 1H). In addition, we found that TP53INP2 could be an independent prognostic factor for the survival of HNSCC (Table 1). Compared with low expression of TP53INP2, those with high expression of TP53INP2 had a significantly lower mortality [0.61 (0.46, 0.81), *P* = 0.0007 in model I; 0.63 (0.47, 0.84), *P* = 0.0016 in model II].

**TABLE 1:** Relationship between TP53INP2 and overall survival of HNSCC.

Outcome	Crude model		Model I		Model II	
	HR (95%)	*P*-value	HR (95%)	*P*-value	HR (95%)	*P*-value
**TP53INP2**						
Low expression	Reference		Reference		Reference	
High expression	0.62 (0.47, 0.82)	0.001	0.61 (0.46, 0.81)	0.0007	0.63 (0.47, 0.84)	0.0016

*Model I adjusted for age and sex. Model II adjusted for age, sex, grade, and stage.*

### Potential Mechanism of TP53INP2 in Regulating HNSCC Progression

**FIGURE 2:**
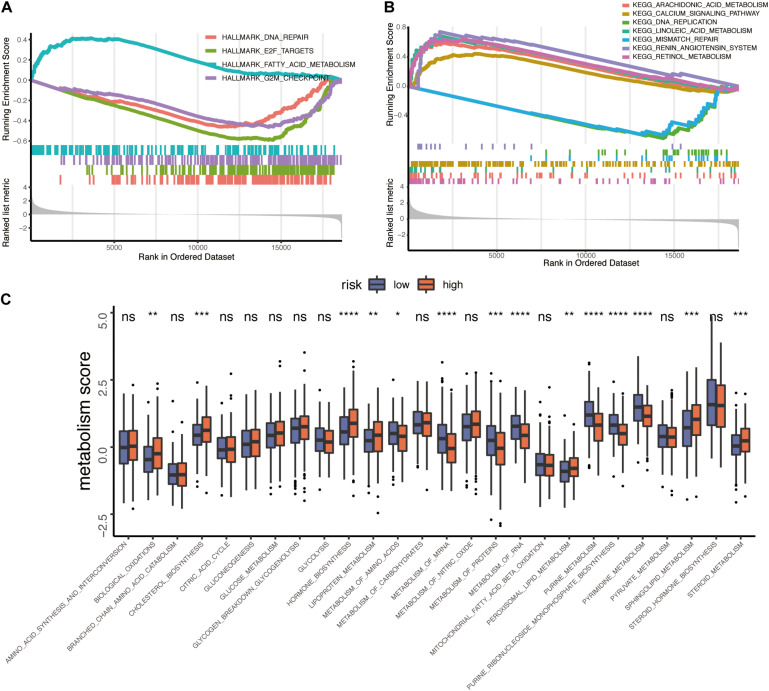
Functional analysis of TP53INP2 in TCGA-HNSC dataset. **(A,B)** GSEA analysis of Hallmarks and KEGG pathway gene sets in TP53INP2-high vs. TP53INP2-low. **(C)** Box plot of 26 metabolism pathways between TP53INP2-high and TP53INP2-low.

To elucidate whether TP53INP2 participates in the progression of HNSCC, we performed the GSEA analysis. The results showed that TP53INP2 was negatively correlated with DNA replication, DNA repair, and cell cycle. On the other hand, TP53INP2 was involved in multiple metabolic pathways, such as lipid metabolism and retinol metabolism (Figures 2A,B). To systematically evaluate the involvement of TP53INP2 in metabolic pathways, we compared the differences in 26 metabolic pathways (Choi and Na, 2018). Biological oxidations, cholesterol biosynthesis, hormone biosynthesis, lipoprotein metabolism, peroxisomal lipid metabolism, and steroid metabolism were significantly up-regulated in TP53INP2-high. Conversely, lower levels of amino acids, mRNA metabolism, proteins metabolism, RNA metabolism, purine metabolism, purine ribonucleoside monophosphate biosynthesis, and pyrimidine metabolism were found in TP53INP2-high (Figure 2C).

**FIGURE 3:**
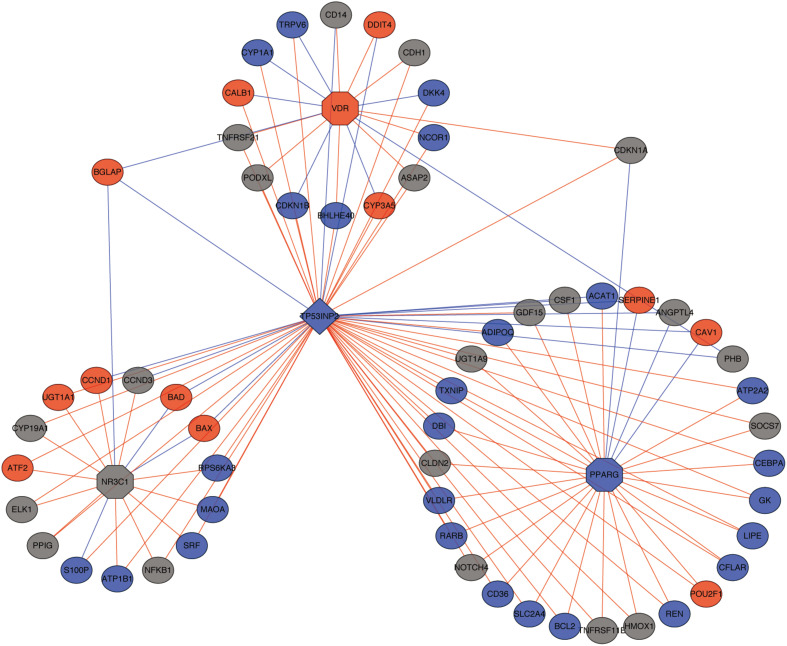
The network of TP53INP2 and targeted genes of nuclear hormone receptors. Red dot represents up-regulated targeted genes; blue dot represents down-regulated targeted genes; red line represents positive correlation; blue line represents negative correlation.

An important function of TP53INP2 is targeting THRA/TRα, GR, VDR, and PPARG to increase their transcriptional activity. Thus, we performed correlation analysis between TP53INP2 and targeted genes of nuclear hormone receptors, as well as the relationship between nuclear hormone receptors and their targeted genes. The related networks were demonstrated in Figure 3 and Supplementary Table 1. Another function of TP53INP2 is participating in autophagy. However, we did not find the difference in autophagy level between TP53INP2-high and TP53INP2-low (Supplementary Figure 1).

### Construction of ceRNA Network Based on TP53INP2 Expression

**FIGURE 4:**
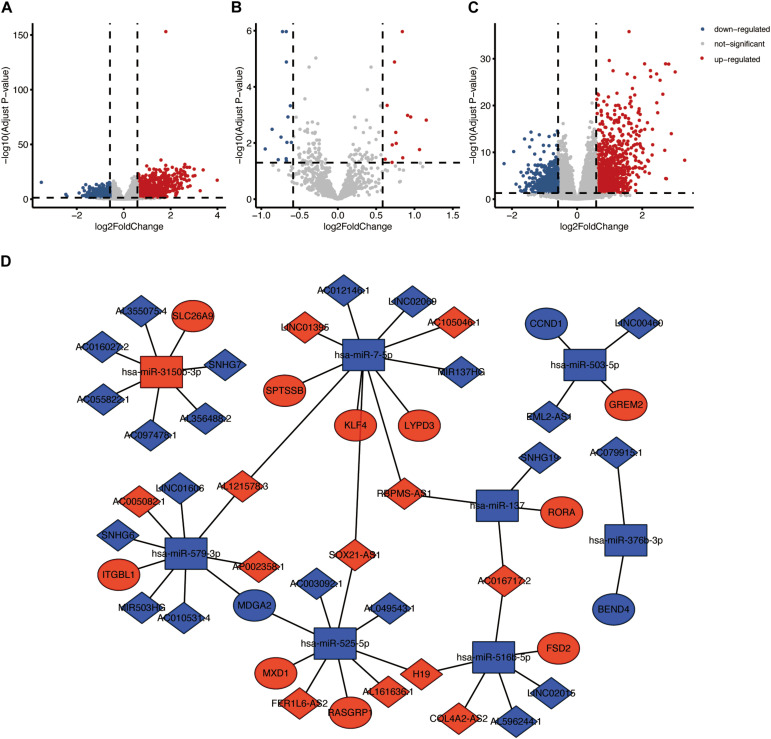
ceRNA network based on the TP53INP2 expression in HNSCC. **(A)** Volcano plot of differentially expressed mRNAs between TP53INP2-high and TP53INP2-low. **(B)** Volcano plot of differentially expressed miRNAs between TP53INP2-high and TP53INP2-low. **(C)** Volcano plot of differentially expressed lncRNAs between TP53INP2-high and TP53INP2-low. **(D)** LncRNAs mediated ceRNA network in HNSCC based on the expression of TP53INP2. LncRNAs, mRNAs, and miRNAs are represented by diamond, ellipse, and rectangle, respectively.

Based on the criteria (|FC| > 1.5 and FDR < 0.05), a total of 1,796 DEmRNAs, 26 DEmiRNAs and 1,827 DElncRNAs were identified (Figures 4A–C). According to the starbase database, we identified 71 lncRNA-miRNA pairs, including 55 lncRNAs and 13 miRNAs. A total of 335 target genes of the above 13 miRNAs were identified. Among 335 target genes, 17 genes were DEmRNAs. We finally constructed a ceRNA network including 33 lncRNAs, eight miRNAs, and 13 mRNAs based on the links of lncRNA-miRNA and miRNA-mRNA (Figure 4D and Supplementary Table 2). The survival-related genes in the ceRNA network were shown in Supplementary Figures 2–4. In addition, the relationship between mRNAs in ceRNA and drugs was presented in Supplementary Figure 5.

### CNV and Mutational Event Analysis in Patients With Different Expression of TP53INP2

**FIGURE 5:**
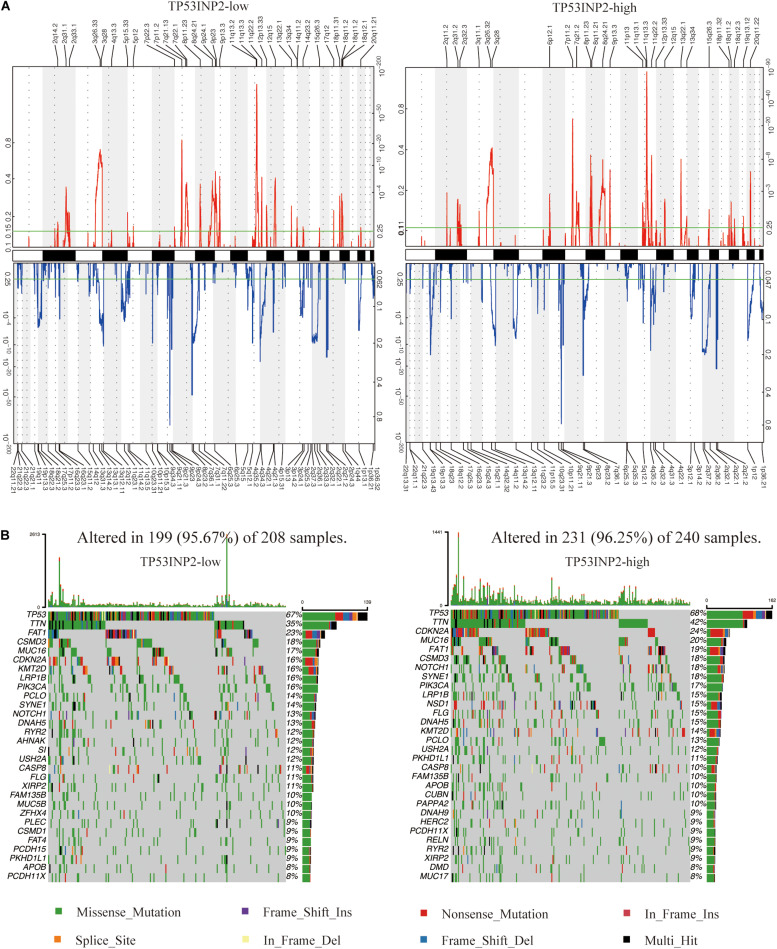
Distinct genomic profiles are correlated with TP53INP2 expression. **(A)** GISTIC 2.0 amplifications and deletions in TP53INP2-high and TP53INP2-low. **(B)** Somatic mutations in TP53INP2-high and TP53INP2-low.

We identified 91 and 67 genomic events enriched in TP53INP2-low and TP53INP2-high based on GSITIC analysis (Figure 5A). In TP53INP2-low patients, oncogenic genes such as EGFR (7p11.2), CDK6 (7q21.13), YAP1 (11q22.2), and MMP7 (11q22.2) were frequently amplified genomic regions, while tumor suppressor genes including CDKN2A (9p21.3), KLF6 (10p15.3), and PTEN (10q23.31) were frequently deleted genomic regions. In TP53INP2-high samples, the two main significant amplification peaks were 11q13.3 and 7p11.2 while 9q21.11 was the most significant deletion peak. Based on TP53INP2 expression levels, we found similar somatic mutation profiles between TP53INP2-high and TP53INP2-low (Figure 5B).

## Discussion

TP53INP2, a protein-coding gene, regulates the transcriptional activity of nuclear hormone receptors, autophagy, rDNA transcription, and ubiquitylation. However, its function in HNSCC is largely unclear. Thus, this study systematically analyzed the value of TP53INP2 in HNSCC.

In this study, we found that TP53INP2 was down-regulated in HNSCC, especially in patients with poor differentiation and lymphatic metastasis. Knockdown of TP53INP2 promoted CAL27 cell proliferation. Correspondingly, low expression of TP53INP2 indicated a poor prognosis. Furthermore, some oncogenic genes like EGFR, CDK6, YAP1, and MMP7 were amplified in the TP53INP2-low patients while some tumor suppressor genes including CDKN2A, KLF6, and PTEN were deleted. These results indicated that TP53INP2 serve as a tumor suppressor gene in HNSCC. However, TP53INP2 acts as an oncogene in bladder cancer evidenced by its promotion of migration and invasion in bladder cancer cells (Zhou et al., 2020). Thus, the function of TP53INP2 might vary from the location of tumors.

Functional analysis suggested that TP53INP2 was associated with multiple metabolism pathways. Among these metabolism pathways, purine ribonucleoside monophosphate biosynthesis was higher in TP53INP2-low and it was reported to be associated with poor prognosis in HNSCC (Choi and Na, 2018). This could partly explain why HNSCC with low expression of TP53INP2 had a shorter survival time. Additionally, it has been found that TP53INP2 negatively regulates adipogenesis in preadipocytes (Romero et al., 2018). In our study, we found that the balance of lipid metabolism was disrupted when TP53INP2 was dysregulated, which might attribute to the altered expression of multiple lipid-related genes (e.g., ADIPOQ, SERPINE1, and DBI) affected by TP53INP2 *via* enhancing the transcriptional activity of PPARG.

Increasing studies have shown that the network mediated by lncRNAs plays an important role in tumorigenesis and progression. Thus, we constructed a ceRNA network based on the high/low expression of TP53INP2. After selecting significant lncRNAs potentially regulated by TP53INP2 expression, we noticed that LINC00460, an oncogene in HNSCC (Jiang et al., 2019; Xue et al., 2019), was also up-regulated in TP53INP2-low patients. Furthermore, we discovered that LINC00460 might regulate CCND1 which also served as an oncogene (Hanken et al., 2014; Gan et al., 2019) and was highly expressed in TP53INP2-low patients as well. Additionally, SOX21-AS1 has been reported to be a tumor suppressor gene in oral squamous cell carcinoma (Yang et al., 2016) and it was up-regulated in TP53INP2-high patients. We found that SOX21-AS1 might control the expression of MXD1, which was a tumor suppressor gene (Wu et al., 2014). However, another oncogene H19 (Guan et al., 2016; Kou et al., 2019) was up-regulated in TP53INP2-high patients, suggesting the potential feedback loop in the HNSCC carcinogenesis of the TP53INP2-high patients regulated by lncRNAs.

## Conclusion

In conclusion, our study highlights the value of TP53INP2 in predicting HNSCC prognosis and its potential function in affecting cell viability, gene expression, metabolism pathways, copy number alteration, and ceRNA network. These results indicate the underlying mechanisms leading to the poor prognosis of HNSCC with lower TP53INP2 expression.

## Data Availability Statement

Publicly available datasets were analyzed in this study. This data can be found here: The Cancer Genome Atlas (TCGA) dataset (https://portal.gdc.cancer.gov/) and Gene Expression Omnibus (GEO) database (https://www.ncbi.nlm.nih.gov/geo/).

## Author Contributions

JX and BC designed the study and supervised the project. RC performed all the bioinformatics analysis described here. SL performed *in vitro* experiments. RC, JZ, and SL wrote and edited the manuscript. JZ, XR, and XC collected and examined the data. All authors read and approved the final manuscript.

## Conflict of Interest

The authors declare that the research was conducted in the absence of any commercial or financial relationships that could be construed as a potential conflict of interest.

## Footnotes

1 https://portal.gdc.cancer.gov/

2 https://www.ncbi.nlm.nih.gov/geo/

3 https://www.grnpedia.org/trrust/

4 http://starbase.sysu.edu.cn/

5 http://gdac.broadinstitute.org

## Supplementary Material

Supplementary Figure 1 Box plot of autophagy level between TP53INP2-high and TP53INP2-low.

Supplementary Figure 2 Survival analysis of mRNAs in TP53INP2 related ceRNA network.

Supplementary Figure 3 Survival analysis of miRNAs in TP53INP2 related ceRNA network.

Supplementary Figure 4 Survival analysis of lnRNAs in TP53INP2 related ceRNA network.

Supplementary Figure 5 The relationship between drugs and mRNAs in TP53INP2 related ceRNA network.

Supplementary Table 1 The coefficients of correlation of Figure 3.

Supplementary Table 2 The coefficients of correlation of Figure 4.
