# Supplementary figures and images for: Integrative Analysis of TP53INP2 in Head and Neck Squamous Cell Carcinoma
Source: Front Genet. 2021 Apr 9;12:630794. doi: 10.3389/fgene.2021.630794 (PMC8062980; doi:10.3389/fgene.2021.630794)

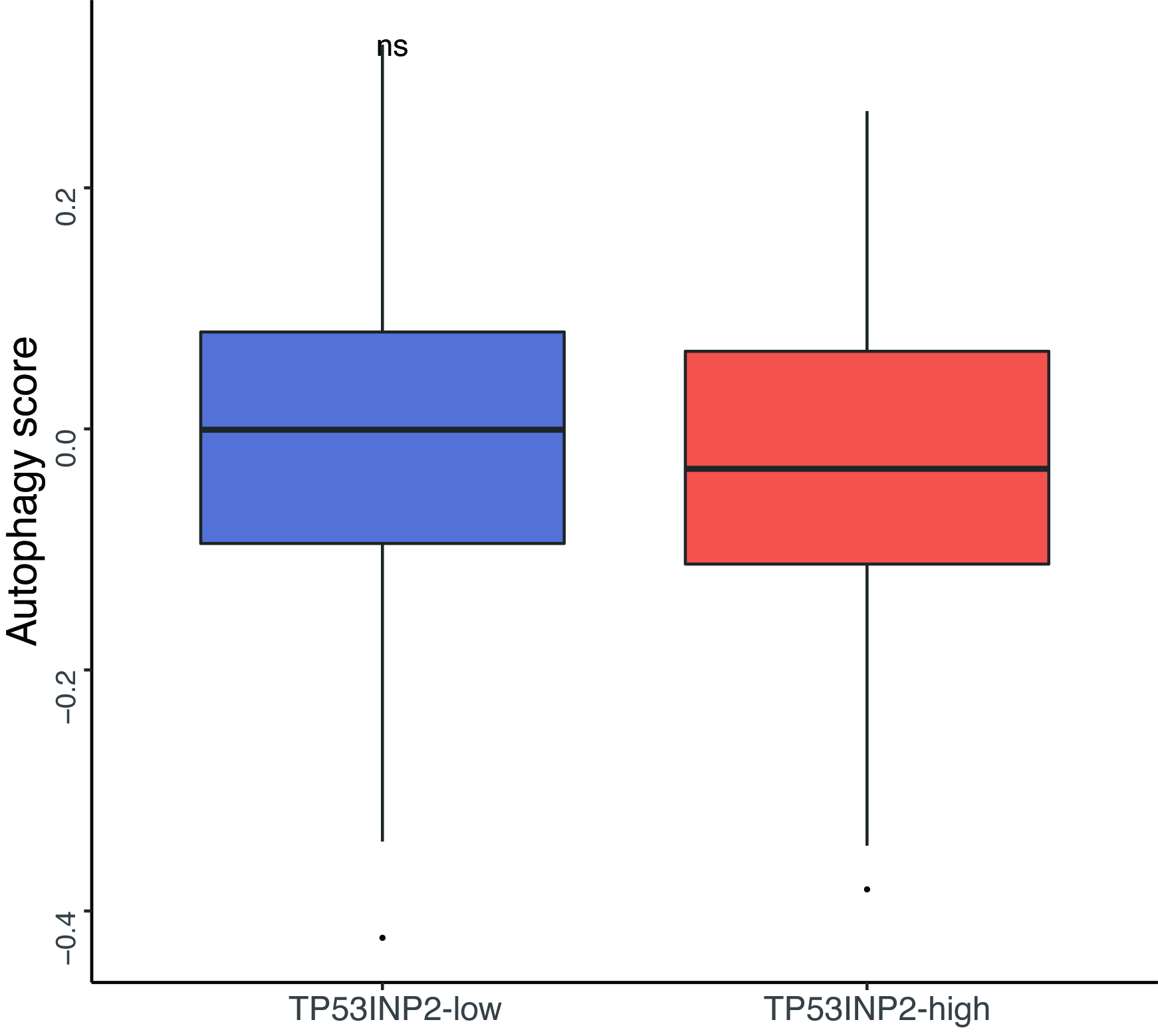

Supplement: Supplementary Figure 1 — Box plot of autophagy level between TP53INP2-high and TP53INP2-low. [file Image_1.TIF]

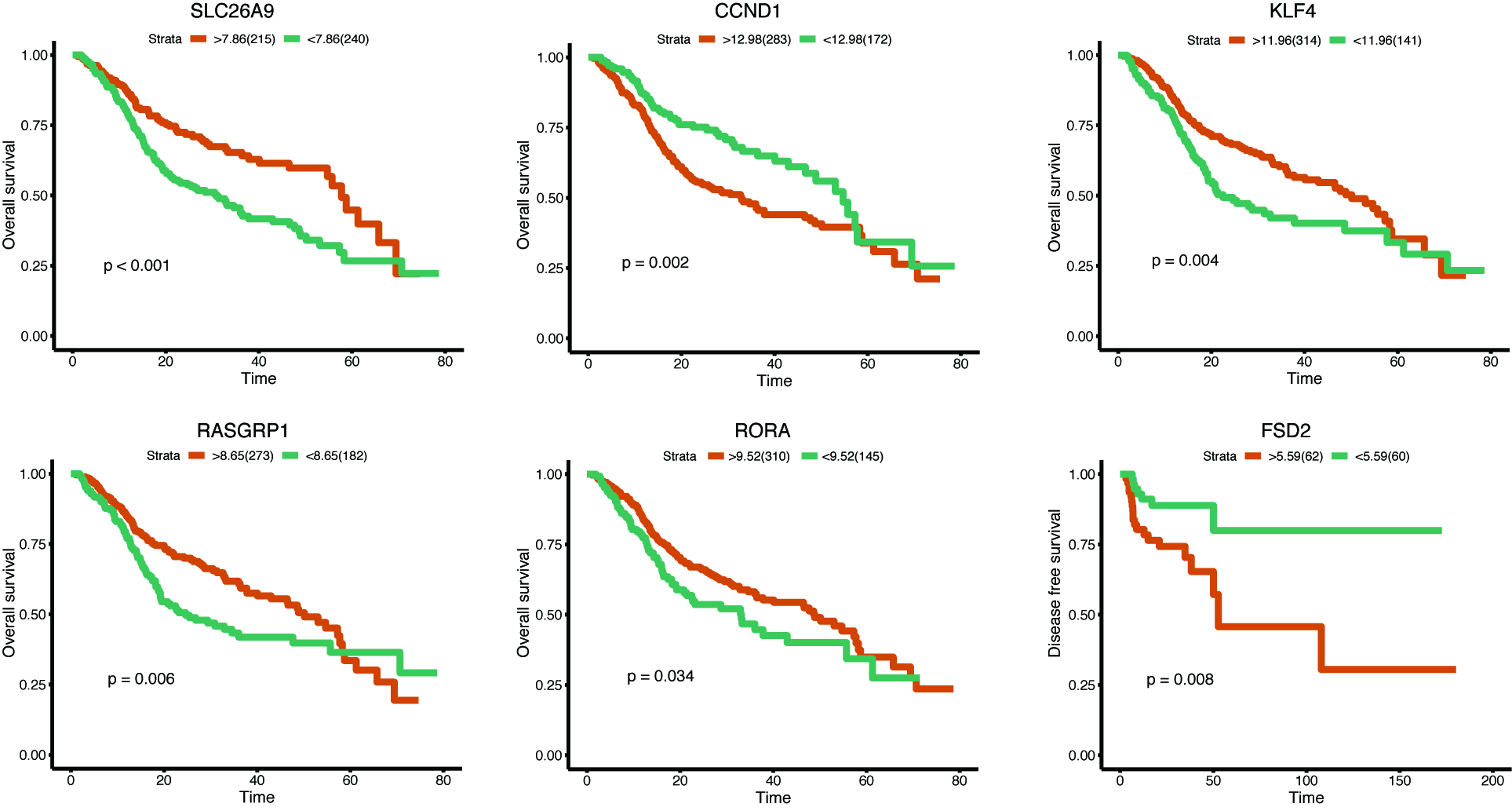

Supplement: Supplementary Figure 2 — Survival analysis of mRNAs in TP53INP2 related ceRNA network. [file Image_2.TIF]

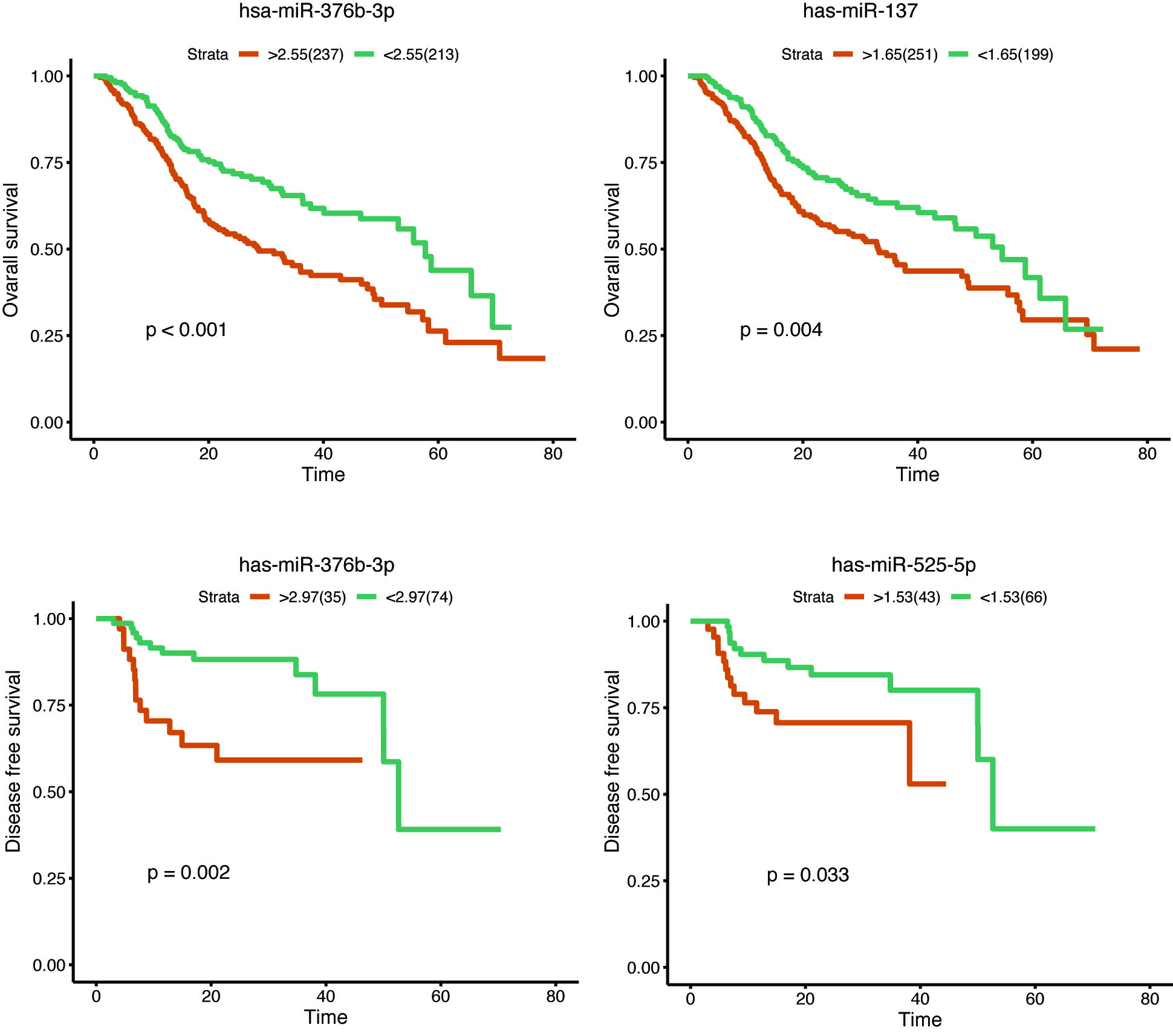

Supplement: Supplementary Figure 3 — Survival analysis of miRNAs in TP53INP2 related ceRNA network. [file Image_3.TIF]

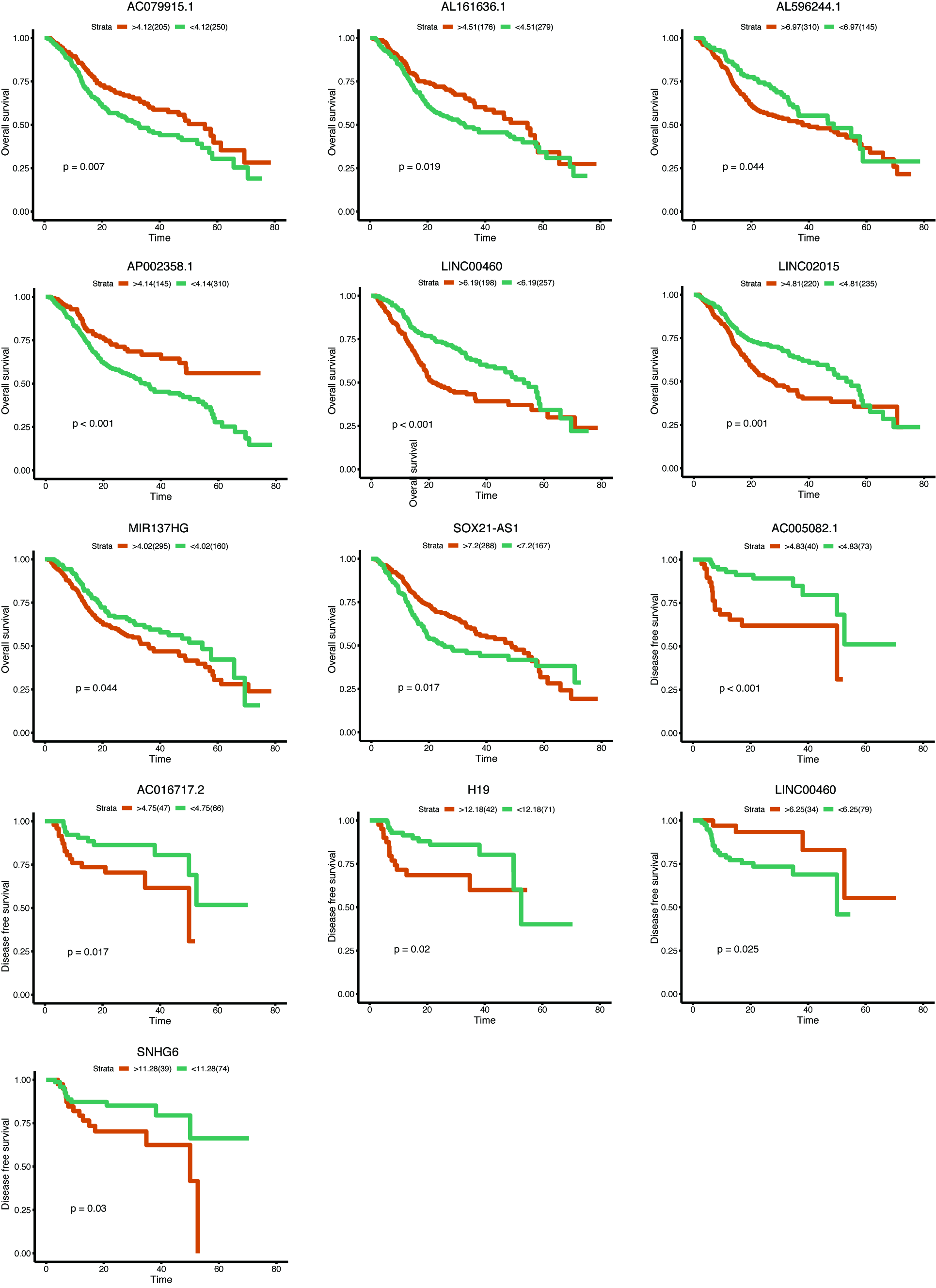

Supplement: Supplementary Figure 4 — Survival analysis of lnRNAs in TP53INP2 related ceRNA network. [file Image_4.TIF]

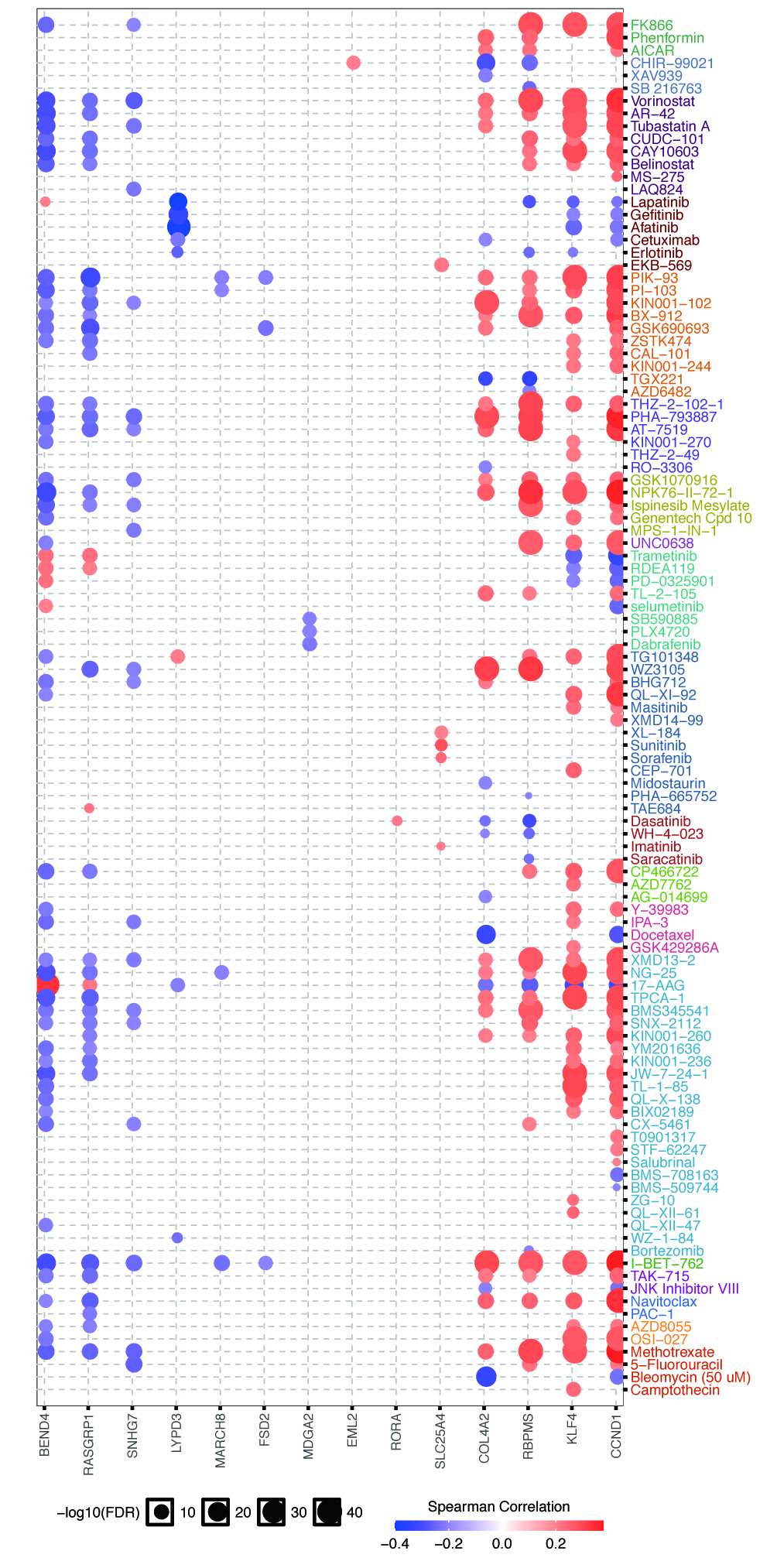

Supplement: Supplementary Figure 5 — The relationship between drugs and mRNAs in TP53INP2 related ceRNA network. [file Image_5.TIF]
